# The Amyloid Assembly of the Bacterial Hfq Is Lipid-Driven and Lipid-Specific

**DOI:** 10.3390/ijms25031434

**Published:** 2024-01-24

**Authors:** Florian Turbant, Quentin Machiels, Jehan Waeytens, Frank Wien, Véronique Arluison

**Affiliations:** 1 Laboratoire Léon Brillouin LLB, CEA, CNRS UMR12, CEA Saclay, 91191 Gif-sur-Yvette, France; florian.turbant@synchrotron-soleil.fr; 2 Synchrotron SOLEIL, L’Orme des Merisiers, Saint Aubin BP48, 91192 Gif-sur-Yvette, France; frank.wien@synchrotron-soleil.fr; 3 Department of Molecular Biology, University of Gdansk, Wita Stwosza 59, 80-308 Gdansk, Poland; 4 Structure et Fonction des Membranes Biologiques, Université Libre de Bruxelles, 1050 Bruxelles, Belgium; quentin.machiels@ulb.be (Q.M.); jehan.waeytens@ulb.be (J.W.); 5 Unit of Pharmacognosy, Bioanalysis and Drug Discovery, Université Libre de Bruxelles, 1050 Bruxelles, Belgium; 6 SDV Department, Université Paris Cité, 75006 Paris, France

**Keywords:** small noncoding regulatory RNA, Hfq, bacterial functional amyloid, protein self-assembly, lipid–protein interaction, cardiolipin, Synchrotron Radiation (Orientated) Circular Dichroism SR-(O)CD

## Abstract

Under specific conditions, some proteins can self-assemble into fibrillar structures called amyloids. Initially, these proteins were associated with neurodegenerative diseases in eucaryotes. Nevertheless, they have now been identified in the three domains of life. In bacteria, they are involved in diverse biological processes and are usually useful for the cell. For this reason, they are classified as “functional amyloids”. In this work, we focus our analysis on a bacterial functional amyloid called Hfq. Hfq is a pleiotropic regulator that mediates several aspects of genetic expression, mainly via the use of small noncoding RNAs. Our previous work showed that Hfq amyloid-fibrils interact with membranes. This interaction influences Hfq amyloid structure formation and stability, but the specifics of the lipid on the dynamics of this process is unknown. Here, we show, using spectroscopic methods, how lipids specifically drive and modulate Hfq amyloid assembly or, conversely, its disassembly. The reported effects are discussed in light of the consequences for bacterial cell life.

## 1. Introduction

When proteins polymerize, they often form structures called amyloids. These structures result from repetitively stacked β-strands (hundreds to thousands), a pattern referred to as a cross-β structure [1]. This results in the formation of unbranched fibrils of ~10 nm in width and up to micrometers in length [2]. In vivo, amyloid fibrils are found in all domains of life, from archaea and bacteria to mammals [3]. The first amyloids were identified in eukaryotes and associated with neurodegenerative diseases, such as Alzheimer’s [4]. Nevertheless, the self-assembled structures may additionally have useful features for the cell; in this case, they are referred to as a “functional amyloid” [5]. These functional roles include biofilm and curli formation in bacteria [6], hormone storage in mammals [7], immune system function in fungi [8] or cell signaling in humans [9]. One main difference distinguishing pathological and functional amyloids is the fact that the latter require the tight regulation of their assembly to avoid the accumulation of species that could be toxic for cells if too abundant. To date, the factors that regulate the assembly and/or disaggregation of the functional amyloids are not well-characterized. In this work, we focus our analysis on the regulation by lipids of the self-assembly of a bacterial functional amyloid, Hfq.

Hfq, standing for Host-Factor Qβ, is a small protein, 102 amino acid residues (aa) in *E. coli*, that forms homohexamers [10]. Present in almost two thirds of bacteria, its function is still unclear in Gram positive bacteria [11]. Conversely, many studies have shown that, in Gram negative bacteria, Hfq is involved in the post-transcriptional control of genetic expression [12]. Hfq precisely controls mRNA translation and degradation using small noncoding RNA (sRNA) [13]. In its soluble form, *E. coli* Hfq presents an intrinsically disordered C-terminal region (CTR) comprising about 38 aa, whereas its N-terminal region (NTR, ~65 aa) adopts an Sm topology [10,14]. The Sm-fold (PROSITE entry PS51536) consists of an α-helix capping a five-stranded bent β-sheet (antiparallel); the β-sheets β4 and β5 from interacting monomers assemble to form the hexameric torus [15,16]. In full-length *E. coli* Hfq, the six CTRs are located at the periphery of the Sm-torus [17,18]. Both faces of the torus, its rim and the CTR are involved in nucleic acid binding, but with different specificities [19,20,21,22]. While the structure of the Sm-NTR is well known, the CTR atomic-structure of *E. coli* Hfq remains unsolved [21,23]. Nevertheless, in some specific conditions, Hfq-CTR folds into an amyloid structure through the formation of a nucleation site of 11 aa [14]. One condition promoting Hfq self-assembly includes the binding to nucleic acids [22]. Note that the presence of this amyloid region seems to be restricted to Gram-negative bacteria harboring a long C-terminus region. Even if analyses of other bacterial Hfq for this property are scarce, β-rich regions possibly form β-amyloid structures, such as that of *Yersinia pseudotuberculosis* CTR, for instance (see https://www.uniprot.org/uniprotkb/B2K204/entry, accessed on 16 January 2024).

Hfq-CTR is essential for Hfq association with lipids and for the promotion of Hfq cluster formation close to the inner membrane (IM) [24,25,26]. Indeed, the membrane-bound form of Hfq has a putative physiological role in RNA metabolism. The partitioning of Hfq in its amyloid form near the IM may allow for the spatial compartmentalization of the protein in the bacteria [26]. This membrane-less segregation, often referred to as a liquid–liquid phase separation (LLPS), is now recognized as one mechanism allowing the formation of membrane-less denser regions, and can involve amyloids [27]. In the case of Hfq, this cellular compartmentalization has been described previously [25,26], and it could play an important role in the control of RNA degradation, for instance by avoiding the premature degradation of mRNAs immediately after transcription [28]. Note that Hfq-CTR has additional cellular roles, including one in DNA compaction [22]. LLPS phase separation using amyloids actually also emerges as a new way of DNA-structuring, as in the case of histone locus bodies [29].

We focus here on how the Hfq amyloid state is markedly influenced by the different lipids found in the *E. coli* inner membrane. There are many examples to suggest that membrane binding is a common property of amyloids and that lipids may influence their aggregation [30]. Still, the lipid composition of the bacteria IM changes throughout bacterial life, and understanding how IM content influences Hfq self-assembly and the reversibility of the process is of fundamental importance to understanding how membranes may dynamically influence Hfq function. Membrane lipid heads may, indeed, influence Hfq–lipid interaction, while the composition of lipid tails influences membrane fluidity, thickness and possible Hfq insertion in the membrane [31,32]. Therefore, *E. coli* may use alterations in IM composition, dependent on growth conditions and the surrounding environment, to change Hfq protein function and location to adapt to stresses [33].

With this work, we intend to analyze the influence of the different lipids found in *E. coli* membranes on Hfq self-assembly, in order to decipher the possible role for Hfq membrane-associated clusters in vivo [28]. The presence of lipidic interfaces, in particular those containing anionic lipids, such as phosphatidylglycerol (PG), phosphatidylserine (PS), phosphatidic acid (PA) or cardiolipin (CL), influences the assembly of pathologic amyloids [34]. In previous works, we have shown that this applies to Hfq-CTR and that *E. coli* membranes (inner IM and outer OM membranes) affect this functional amyloid assembly [32,35]. Here, using Synchrotron Radiation Circular Dichroism (SRCD) and Attenuated Total Reflection Fourier Transform Infrared Spectroscopy, we intend to determine if these effects are lipid-specific. To specify, we used Orientated Circular Dichroism (OCD) spectroscopy to overcome the effects of the alignment of amyloid fibers on CD spectra [36].

## 2. Results

### 2.1. Effects on Hfq-CTR Fibers Are Lipid-Specific

To observe the effect of the lipidic anionic interfaces on the amyloid self-assembly strengthening or disassembly, we used a solution with an intermediate polymerization state (Supplementary Figure S1). Indeed, we already observed that Hfq self-assembly is dynamic and the starting point from partially or fully polymerized states could greatly influence the evolution of the assembly. Fully assembled CTR, for instance, only allows disassembly [14].

**Figure 1:**
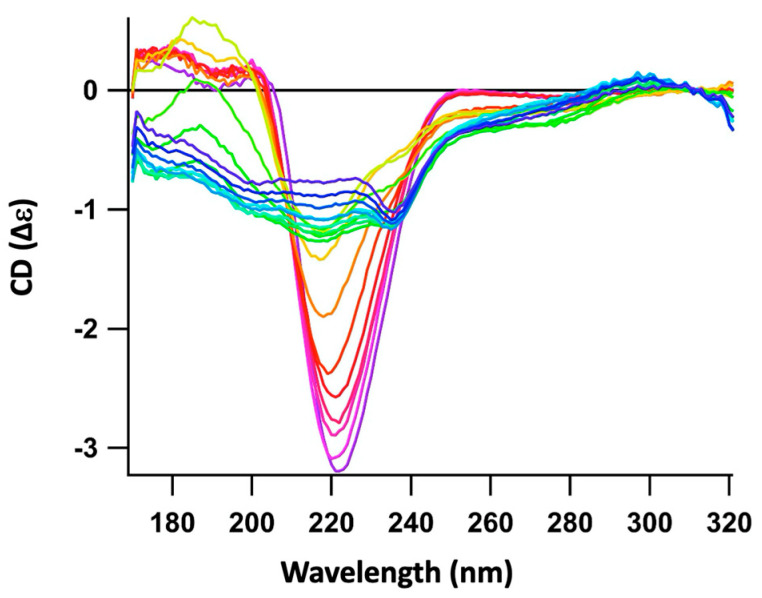
Orientated Synchrotron Radiation Circular Dichroism (OCD) kinetics recorded for ~10 h, demonstrating the evolution of both (i) the interaction between pre-polymerized Hfq-CTR and supported CL bilayers and (ii) the evolution of the CTR structure. Spectra go from blue to green, yellow red and purple, with an interval of ~45 min between each spectrum. The peak corresponding to the amyloid structure is observed around 220 nm.

**Figure 2:**
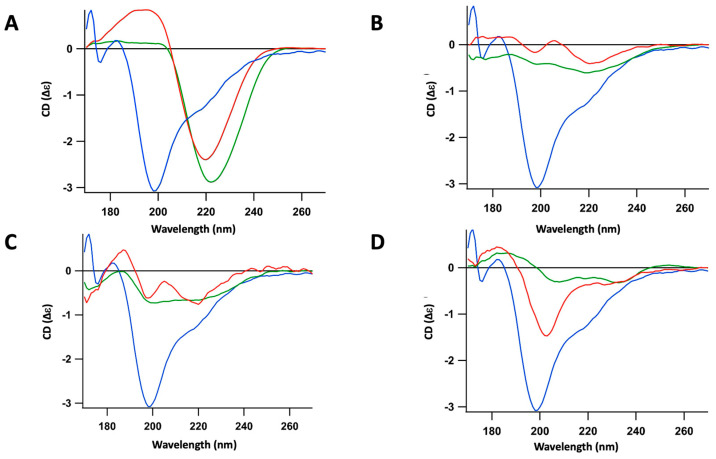
Synchrotron Radiation Circular Dichroism (OCD) analysis of the interaction between the partially polymerized Hfq-CTR (prepared as described in the Section 4) and supported lipidic bilayers. We used Orientated Circular Dichroism (OCD) spectroscopy to overcome the effects of the alignment of amyloid fibers in the CD spectra, which distort the measurement due to a linear dichroism signal [36]. Blue: CTR alone; green: CTR in contact with bilayer for 300 min; red: CTR in contact of bilayer for 400 min after washing to remove CTR unbound from lipid bilayers. (**A**) CL bilayer; (**B**) PG bilayer; (**C**) PE bilayer; (**D**) PA bilayer. The peak corresponding to unstructured peptide is observed around 200 nm, while the peak corresponding to the amyloid structure is observed around 220 nm.

Using OCD, we show that cardiolipin (CL) is the lipid that interacts the most with CTR fibers (Figure 1 and Figure 2A). To be more specific, we observed, after 7 h of interaction with CL, that 50 to 70% of pre-polymerized CTR remained associated with the CL-bilayer (Figure 2A). Furthermore, we observed that, quickly after interaction with the CL bilayer, the peptide self-assembly was strengthened, increasing the characteristic signal of the amyloid structure around 220 nm (Figure 1). With phosphatidylglycerol (PG), only a slight increase in the amyloid structure was observed and two populations, amyloid and unstructured, coexist (Figure 2B). This could be explained by the low amount of peptide retained on the PG bilayer compared with CL. With phosphatidylethanolamine (PE, Figure 2C), the most abundant lipid in the *E. coli* IM, an interaction similar to that with PG occurs. Finally, we observed a moderate interaction with phosphatidic acid (PA) and possibly a slight tendency to disassemble the amyloid fibers towards unstructured peptides (Figure 2D). Note that PA lipids are scarce in the *E. coli* membrane (<1%) and this effect should not be notable in vivo.

**Figure 3:**
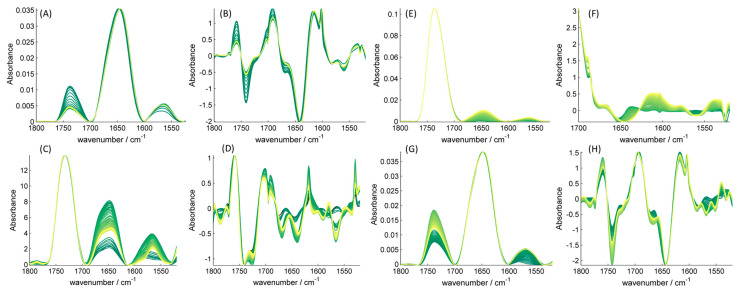
FTIR spectrum of Hfq-CTR monomer in interaction with the different lipids. The spectra presented are: (**A**) with CL, (**C**) DOPG, (**E**) DOPE and (**G**) with DOPA. The second derivative are shown in (**B**) with CL, (**D**) DOPG, (**F**) DOPE and (**H**) DOPA. The spectra are represented from dark-green for the first spectrum to yellow for the last one. We can observe a shift of the maximum of amide I band from 1643 to 1640 cm^−1^ (indicating the formation of intramolecular β-sheets), but we cannot conclude for a clear amyloidogenesis of CTR in contact with specific lipids (indicated by a peak at 1615 cm^−1^).

We then intended to confirm these results using FTIR spectroscopy. To demonstrate the self-assembly of β-sheets in contact with the membrane, lipids were first deposited on an Attenuated Total Reflection (ATR) diamond crystal and dried to evaporate chloroform. On top of the lipid, the protein solution was added in deuterated buffer, and the kinetics were followed by taking IR spectra every 10 min (Figure 3).

With ATR-FTIR (Attenuated Total Reflection Fourier Transform Infrared spectroscopy) the penetration depth is around 1 μm; therefore, only molecules close to the crystal are detected in an ATR setup and only protein species that sediment on the lipids can be detected and analyzed. For this reason, we always observe an increase in the amyloid peak at ~1610 cm^−1^ at the beginning of the kinetics measurements, even in the absence of lipids. This increase is explained by the sedimentation of the fibrils reaching the ATR crystal and not by amyloid self-assembly. ATR-FTIR was thus less suited to quantify CTR assembly in contact with the bilayer, particularly for short times less than 100 min.

### 2.2. Lipids Specifically Induce Amyloid Assembly from Its Soluble Form

**Figure 4:**
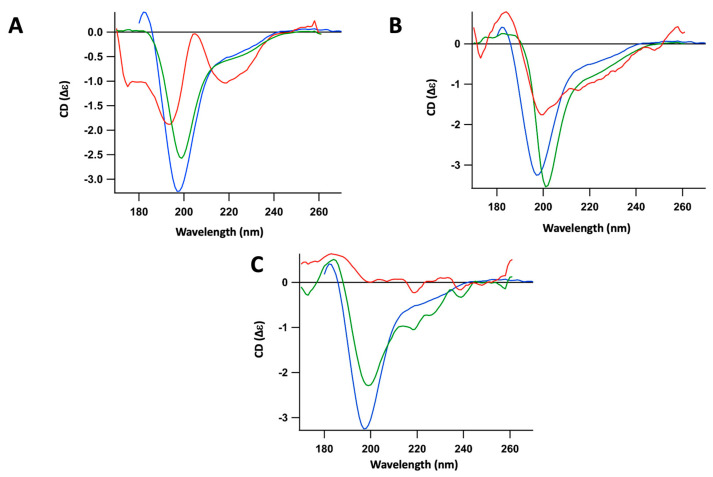
Synchrotron Radiation Circular Dichroism (OCD) analysis of the interaction between soluble Hfq-CTR (prepared extemporaneously just before analysis) and supported lipidic bilayers. Blue: CTR alone; green: CTR in contact with bilayer for 400 min; red: CTR in contact with bilayer for 400 min after washing to remove CTR unbound to the bilayer. (**A**) PG bilayer; (**B**) CL bilayer; (**C**) PE bilayer. The same result was observed for PE and PA; no peptide is retained after washing (in red), indicating very low, if any, interaction.

Next, we analyzed the effect of these various lipids on the efficiency of Hfq-CTR self-assembly from its soluble form (Figure 4). For this, OCD was also used as described in the Section 4. In this case, we observed that PG induced a slight CTR amyloidogenicity, with an increase and a shift of the peak corresponding to unstructured peptide to the red (bathochromic, from 195 nm to 200 nm). After 7 h of incubation and washing to remove unbound species, the emergence of a second population, corresponding to the amyloid structure (~220 nm), was observed (Figure 4A). Note that both the amyloid and unstructured forms co-exist and remain attached to the membrane after washing. For CL, opposite to what we observed with fibers, a low amount of soluble CTR remained attached to the membrane after washing and no clear amyloid structure formation occurred in this case (Figure 4B). Finally, almost no soluble CTR remained attached to the PE and PA membrane after washing (Figure 4C).

We also intended to confirm these results with soluble CTR using FTIR spectroscopy. Nevertheless, the amount of peptide in contact with lipids detected using the ATR was low and the results were difficult to interpret in the monomeric form. In particular, it was not possible to wash unbound CTR in ATR-FTIR to observe the presence of fibers as with SR-OCD.

### 2.3. Phosphatidic Glycerol (PG) Significantly Interacts with Full-Length Hfq

**Figure 5:**
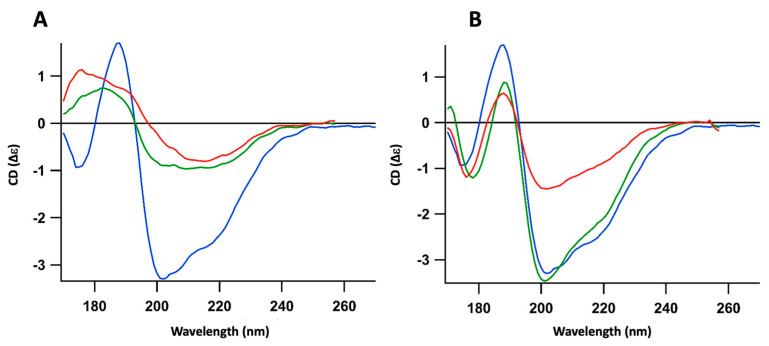
Synchrotron Radiation Circular Dichroism (OCD) analysis of the interaction between full-length Hfq and supported lipidic bilayers. Blue: Hfq; green: Hfq in contact of bilayer for 400 min; red: Hfq in contact of bilayer for 400 min after washing to remove Hfq unbound to bilayer. (**A**) PG bilayer; (**B**) CL bilayer.

Finally, we analyzed the influence of lipids that have the most significant effect on the structure of full-length Hfq and its self-assembly in order to confirm previous results (Figure 5). In this case, we observed that PG induced a slight amyloidogenicity of the full-length protein, with a partial red-shift of the peak from 200 nm to 220 nm (both peaks co-exist and partially overlap). No clear effect was observed with CL. It should be noted, however, that, in the full-length protein, the amyloid structure represents only a minor part of the β-sheets found in the whole protein, as the N-terminus region NTR exists as the β-rich Sm-fold [14,37]; the apparent lack of CL-effect could thus be masked by Hfq-NTR β-sheets.

## 3. Discussion

A growing number of studies indicate that an interaction between lipid bilayers and amyloid-forming proteins occurs and that lipids play an important role in the kinetics of amyloid assembly or stability [38,39,40,41,42,43]. The lipid membrane may, indeed, provide an interface to increase the protein local concentration, facilitating fibrillization. On the other hand, lipids may also destabilize and resolubilize amyloid fibers into a soluble form [44]. Taken together, these results suggest the balance between the soluble and aggregated forms of amyloid-prone proteins is actively determined by compounds found in the cell. The role of lipids could explain why a functional amyloid forms or dissociates rapidly in vivo (when amyloid aggregation is usually a slow process). Nevertheless, such a regulation also implies tight aggregation control (including controlling protein charge, hydrophobicity or concentration) or the use of other molecules, such as other proteins, to control amyloidogenesis [26]. Such control induces a rapid, focused and dynamic aggregation. Our study offers a lipid-mediated explanation for this rapid spatiotemporal regulation in response to environmental changes. Note that pathological amyloid assembly is probably a less controlled process.

With this work, we show that the nature of lipids greatly influences Hfq self-assembly, shedding new light on how lipids may influence Hfq clustering in the vicinity of the inner bacterial membrane [28]. Indeed, in *E. coli*, an important part of Hfq, is found in close proximity to the inner membrane [45]. Nevertheless, the influence of the lipids’ nature on this spatial segregation has never been analyzed before. An *E. coli* membrane is mainly composed of four lipids: PE (~75%), PG (~20%), CL (~5%) and traces of PA. PE is neutral and usually found at around 75% in the IM. CL is negatively charged and represents ~5% of IM lipids during the exponential phase of growth, but increases to 15–20% during the stationary phase [46,47]. PG is negatively charged and comprises 15–20% of *E. coli* lipids during the exponential phase of growth, but only ~5% during the stationary phase [46,47]. Finally, only traces of PA (<1%) are found in the IM. The lipid composition of IM thus significantly changes with the bacterial phase of growth, basically with an increase in CL and decrease in PG. Here, we show that CL boosts the stability of preformed Hfq-CTR fibers, while PG induces CTR amyloid formation from the soluble (monomeric) form of the protein. Furthermore, Hfq concentration also increases during the stationary phase of growth [45], an effect that would also favor its aggregation. Interestingly, the lipids that interact the most with Hfq-CTR (soluble or amyloid forms) are both negatively charged. This interaction could thus be due to residues found in amyloids that are usually hydrophilic and charged (mainly lysines or arginines and glycines, that are present in Hfq and its CTR, see Section 4.1) [48]. These residues may constitute a tag that can sequester the amyloid sequence to maintain the soluble form of the protein, and this tag may be hidden by the lipid charges, thus favoring amyloid self-assembly when the protein is in contact with the membrane.

Previous analyses of amyloids associated with human diseases show that they are often associated with lipid rafts [30]. If lipid rafts do not exist in bacteria, prokaryotes have Functional Membrane Microdomains (FMM), which are equivalent to rafts [49]. These bacterial microdomains influence bacterial signal transduction, protein secretion or transport across membrane [50,51]. CL, with its larger structure, is the major component of these FMMs and CL-rich microdomains; in particular, it can occur at the cell poles in *E. coli*, but also at the division septum [51,52]. The dense nature of FMM probably reduces the diffusion of associated proteins, which facilitates protein assembly. The ability of CL to strengthen the amyloid-like fibers around FMMs may thus provide a mechanism for the partitioning of Hfq into clusters near the IM and could influence the RNA-related process of regulation [25,28]. The sequestration of Hfq to the pole may additionally depend on other proteins, such as the pole-localizer TmaR [26,53]. Furthermore, CL has an important role in modulating the membrane composition to adapt to stress conditions and to change membrane fluidity [54,55]. This function could be linked to the role of Hfq in answering numerous stresses [56,57]. Such a CL-specific effect could eventually be tested in vivo using a strain devoid of CL-synthase (the activity of which, in turn, increases significantly during the stationary phase in the Wild-Type strain) [58].

Another particularity of CL is its effect on membrane thickness. *E. coli* IM thickness is around 3.7 nm [59], while the thickness of pure PG membranes is ~3.6 nm. When CL is inserted in a PG membrane, its thickness increases to ~3.8–3.85 nm [60]. CL additionally modifies lipid ordering in the bilayer [60]. These effects are mainly due to the higher volume of CL compared with PG and PE; the CL small head (glycerol bridge) with its hydrophobic body composed of four fatty acids gives it a conical shape compared with the cylindrical shape of PG [61]. Due to this shape, CL induces curved regions within the IM [62]. The formation of curved CL-rich domains may help to locate the cell division septum and, in turn, play an important role in attracting the DNA-bound amyloid form of Hfq [22] for chromosome partitioning after replication [63,64]. Note that CL also accumulates in *E. coli* minicell membranes [65] and that Hfq influences the formation of these minicells [66,67]. Finally, it has also been shown that the lifetimes of pores formed in CL-containing membranes decreased compare to CL-free bilayers [60]. This could also influence the lifetime of pores formed by Hfq in IM [32].

**Figure 6:**
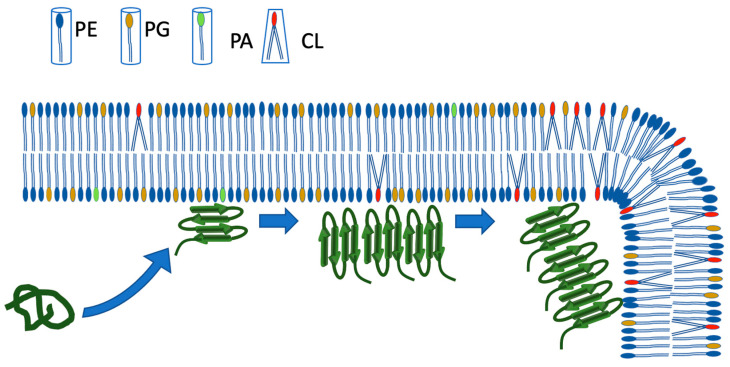
Model of Hfq-CTR interaction with *E. coli* membrane. For simplification, only the CTR of Hfq is represented, not the Sm torus. First, the soluble form of Hfq interacts with the IM. PG is randomly distributed in the IM and induces amyloidogenicity of the CTR. Then, Hfq fibers interact with CL-rich FMM microdomains. This strengthens self-assembly. Note the conical shape of CL due to its four fatty acyl side chains, compared to the cylindric shape of PG, PE and CL. The interaction with CTR and polar lipids could possibly induce a phase separation of PE and favor the formation of FMM [32]. As CL microdomains are found at the pole of the cell, and due to the increased concentration of CL during stresses and the stationary phase [54,55], the migration of Hfq to the poles during stresses could be due to its interaction with CL [26]. As shown previously, PG may destabilize the fiber and promote Hfq insertion into the membrane at a later stage; this insertion may induce the formation of pores in the IM and Hfq translocation into the periplasm [14,32,35]. Taking into account CL properties [60], this should, rather, occur in PE/PG-rich regions.

As for the effect of PG on amyloid assembly, here we show that PG induces CTR amyloidogenicity, particularly from its soluble form, while CL has the main effect on preformed fibers. Both lipids are negatively charged but, in contrast to CL, PG has not been reported to form membrane microdomains. One important difference between CL and PG is that PG is randomly distributed in the IM, while CL concentrates at the cell poles. Taken together, these results suggest that PG could help the random nucleation of CTR amyloid form from the soluble form in the proximity of the IM, without a specific location. This allows the formation of Hfq foci that may migrate to the cell poles when stresses occur as well as IM composition and structure changes (Figure 6). As the Hfq C-terminal amyloid region lacked a clear function for years, its specific interaction with bacterial membranes [24,32,45] opens up new and unexpected ways to regulate the function of this master regulator by spatially trapping it into membrane-associated clusters [25,28]. The lipid specificity governing Hfq amyloidogenesis—or dissociation—that we evidence here suggests that lipids may guide Hfq cellular relocation during the cell cycle. This could, in particular, explain Hfq migration from forming extended fibers coiling along the axis of the cell under normal conditions of growth to the cell pole patches under stresses conditions [26]. This cellular compartmentalization could ultimately play an important role in controlling sRNA-dependent pathways to adapt to stress conditions [33].

## 4. Materials and Methods

### 4.1. Preparation of Hfq and Hfq-CTR

The sequence of the Hfq-CTR peptide was SRPVSHHSNNAGGGTSSNYHHGSSAQNTSAQQDSEETE. This sequence corresponds to residues 64 to 102 in full-length Hfq protein. *E. coli* Hfq-CTR peptide was chemically synthetized (Proteogenix, Strasbourg, France) and prepared at 20 mg/mL. In order to observe the effect of lipids on both polymer strengthening and disassembly, we used a solution with an intermediate polymerization state. To specify, Hfq CTR was incubated for one week at 20 °C or put in solution extemporaneously just before use. The partial formation of the amyloid structure for intermediate polymerization was confirmed using FTIR spectroscopy as shown on Supplementary Figure S1.

The wild-type *E. coli* Hfq protein was purified following the previously described protocol [24] at a concentration of 8 mg/mL. Note that, at this concentration, the soluble form of the protein corresponds to Hfq hexamers (possibly dimers of hexamers can occur [68]); no monomer can be detected [69].

### 4.2. Preparation of Small Unilamellar Vesicles (SUVs)

The lipids used in this study were from Avanti Polar Lipids (Alabaster, AL, USA) and were dissolved and conserved at 10 mg/mL in a solution of chloroform:methanol 1:1 (*v*/*v*) on ice. The lipids used were 1,2-dioleoyl-sn-glycero-3-phosphoethanolamine (DOPE), 1,2-dioleoyl-sn-glycero-3-phosphate (DOPA), cardiolipin (CL) and 1,2-dioleoyl-sn-glycero-3-phospho-(1′-rac-glycerol) (DOPG), the components of *E. coli* IM [31]. For brevity, we refer to DOPE as PE, DOPA as PA and DOPG as PG throughout this paper. The solvent was evaporated under a N_2_(g) flow; after 30 min of nitrogen flow, a lipid film formed. Then, SUV buffer (10 mM sodium phosphate buffer pH 7.5 containing 100 mM NaCl) was added followed by slow stirring for 30 min at room temperature to rehydrate the lipids and to form the vesicles. To obtain homogeneous Small Unilamellar Vesicles (SUVs), the mixture was passed about 30 times through a 0.1µm polycarbonate filter using an extruder (Avanti Polar Lipids, Alabaster, AL, USA). The SUV samples were conserved at 4 °C for a maximum of 1 week.

### 4.3. Preparation of Lipid Supported Membranes for OCD Analysis

For the formation of supported membranes, CaCl_2_ at a final concentration of 2 mM was added to the SUVs solution. A total of 20 µL of the mix was put on a CaF_2_ cell surface and incubated for 1 h at RT. Then, the surface was washed with dd water five times to remove the excess lipids. The lipid membrane was always kept in a moisturized chamber with a saturated K_2_SO_4_ solution providing a humidity of 97%. Pure cylindric lipid (PG, PE) or conic lipid (CL) bilayers should adopt a structure close to that reported in Sennato et al. [70].

### 4.4. Synchrotron Radiation Circular Dichroism (SRCD) and Orientated Circular Dichroism (OCD)

The OCD measurement and data analysis were carried out on the DISCO beamline at Synchrotron SOLEIL (proposal 20220056 and 20230530). SUVs were deposited on the CaF_2_ cell surface (4 µL). Lipid films must be thin to minimize scattering and assure homogeneity. A volume of 1 µL of Hfq (8 mg/mL) or CTR (20 mg/mL) solutions were then dispensed on top. A CaF_2_ lid was put on top (14 µm pathlength), preserving the humidity within the closed cell and keeping the membrane/protein sample airtight. Spectral acquisitions of 1 nm steps between 180 and 270 nm were recorded at a temperature of 20 °C. The OCD spectra were averaged by recording CD spectra of the beam-centered cell, with the cell turning clockwise 45° using an automated rotating chamber. This method allows the elimination of a linear dichroism signal due to the alignment of the peptide, resulting in a deformed CD spectrum. Relevant experiments, where an effect can be observed, were repeated twice to ensure their reproducibility. Data analysis, including averaging, baseline subtraction, smoothing, scaling and standardization, was carried out with CDtoolX [71].

### 4.5. FTIR Analysis

Attenuated total reflection Fourier transform infrared spectroscopy (ATR-FTIR) measurements were obtained with a Bruker Equinox55 spectrometer (Billerica, MA, USA) purged with dry air and a diamond ATR device with a single reflection at an angle of 45° and closed with a golden gate chamber from Specac (Orpington, UK). A total of 5 µL of Hfq-CTR solution at 20 mg/mL in D_2_O was deposited. The data were treated with kinetics, a custom-made program (SFMB, Université libre de Bruxelles, Bruxelles, Belgium) running under Matlab 7.5.0 (Mathworks, Natick, MA, USA). The water vapor signal was removed by subtracting a reference spectrum of pure water vapor with a coefficient optimized on the amide II area band (1555–1550 cm^−1^). To analyze the interaction with lipids, a drop of lipid at 10 mg/mL was deposited on the ATR surface and dried with a nitrogen flux. Then, 5 µL of Hfq-CTR solution at 20 mg/mL was deposited on the lipids. A series of spectra with a resolution of 4 cm^−1^ were acquired every 10 min overnight.

## Data Availability

The data that support the findings of this study are available on request from the corresponding authors.

## Supplementary Materials

The following supporting information can be downloaded at: https://www.mdpi.com/article/10.3390/ijms25031434/s1.
