# Supplementary material for: The Amyloid Assembly of the Bacterial Hfq Is Lipid-Driven and Lipid-Specific
Source: Int J Mol Sci. 2024 Jan 24;25(3):1434. doi: 10.3390/ijms25031434 (PMC10855545; doi:10.3390/ijms25031434)
Supplement: Supplementary file 1 [file ijms-25-01434-s001.zip › ijms-2821378-supplementary.pdf]

Supplementary Materials

The Amyloid Assembly of the Bacterial Hfq Is Lipid-driven and Lipid-specific

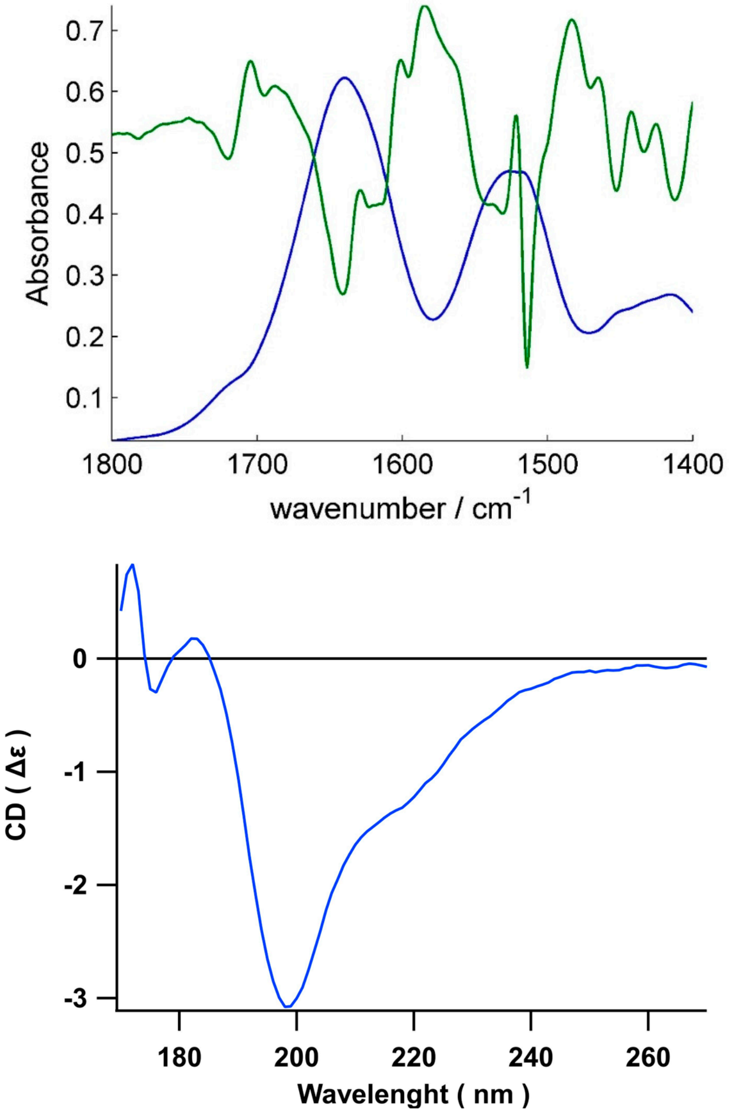

Figure S1: ***Top:*** FTIR spectrum of Hfq-CTR with an intermediate polymerization state in Blue and the 2nd derivative of this spectrum in green. The shoulder observed around 1615 cm^{-1} in the FTIR spectrum is confirmed in the 2nd derivative where we observe a clear minimum this frequency. It is typical for intermolecular β-sheet structure observed in amyloid fibrils. The band is weak indicating an intermediates aggregation compare to fully polymerized spectrum available in previous work (Waeytens et al, 2021 doi:10.1039/d0an01545h); ***Bottom:*** SRCD spectrum of the same solution of Hfq-CTR peptide, where the presence of the amyloid form is indicated by the shoulder ~ 220 nm.
